# Gray matter, white matter and cerebrospinal fluid abnormalities in Parkinson’s disease: A voxel-based morphometry study

**DOI:** 10.3389/fpsyt.2022.1027907

**Published:** 2022-10-17

**Authors:** Charles Okanda Nyatega, Li Qiang, Mohammed Jajere Adamu, Halima Bello Kawuwa

**Affiliations:** ^1^School of Electrical and Information Engineering, Tianjin University, Tianjin, China; ^2^Department of Electronics and Telecommunication Engineering, Mbeya University of Science and Technology, Mbeya, Tanzania; ^3^School of Microelectronics, Tianjin University, Tianjin, China; ^4^Department of Biomedical Engineering, Tianjin University, Tianjin, China

**Keywords:** Parkinson’s disease, MRI, voxel-based morphometry, gray matter, white matter, cerebrospinal fluid, statistical parametric mapping (SPM12)

## Abstract

**Background:**

Parkinson’s disease (PD) is a chronic neurodegenerative disorder characterized by bradykinesia, tremor, and rigidity among other symptoms. With a 70% cumulative prevalence of dementia in PD, cognitive impairment and neuropsychiatric symptoms are frequent.

**Materials and methods:**

In this study, we looked at anatomical brain differences between groups of patients and controls. A total of 138 people with PD were compared to 64 age-matched healthy people using voxel-based morphometry (VBM). VBM is a fully automated technique that allows for the identification of regional differences in gray matter (GM), white matter (WM), and cerebrospinal fluid (CSF) allowing for an objective comparison of brains of different groups of people. We used statistical parametric mapping for image processing and statistical analysis.

**Results:**

In comparison to controls, PD patients had lower GM volumes in the left middle cingulate, left lingual gyrus, right calcarine and left fusiform gyrus, also PD patients indicated lower WM volumes in the right middle cingulate, left lingual gyrus, right calcarine, and left inferior occipital gyrus. Moreover, PD patients group demonstrated higher CSF in the left caudate compared to the controls.

**Conclusion:**

Physical fragility and cognitive impairments in PD may be detected more easily if anatomical abnormalities to the cingulate gyrus, occipital lobe and the level of CSF in the caudate are identified. Thus, our findings shed light on the role of the brain in PD and may aid in a better understanding of the events that occur in PD patients.

## Introduction

Parkinson’s disease (PD) is a degenerative neurological condition that is chronic and progressive that mainly affects the motor system. It is the second most common neurodegenerative disease after Alzheimer’s disease and its prevalence is predicted to rise as the population ages (1) with 1% of adults over the age of 65 affected (2). PD is characterized by the degeneration of dopamine neurons in the substantia nigra (SN) of the midbrain, with concomitant loss of their axons that project to the striatum along the nigrostriatal pathway. This results in loss of the neurotransmitter dopamine which leads to the primary motor symptoms of PD (3), which were first described by James Parkinson in 1817 as a heterogeneous manifestation (4). Currently, it is thought that oxidative stress, mitochondrial malfunction and protein mishandling play a key part in the pathogenesis of PD (5). The etiology of the disease is unknown, however, both genetic and environmental factors are thought to have a role with males more likely than females to be affected at a ratio of around 3:2 (6), for example, estrogen and oxitocin, which are predominantly female neurotransmitters and hormones, serve as a protective mechanism for the female nervous system (7). Rigidity, tremor, slowness of movement (bradykinesia), and postural instability are considered cardinal indicators of PD (8), which present themselves clinically once the levels of striatal dopamine decrease by 70% (9). Rigidity is an increased muscular tone, or a constant and excessive contraction of muscles, causes stiffness and resistance to limb movement. It might be homogeneous (“lead-pipe rigidity”) or ratchet-y (“cogwheel rigidity”) (8, 10), often coupled with joint pain, which is a common first symptom of the disease. A tremor symptom is the tendency of the index finger and thumb to contact and make a circular movement simultaneously. Bradykinesia is accompanied with difficulties throughout the movement process and it makes it impossible to do two separate motor actions at the same time. While most clinicians can detect bradykinesia, a formal assessment requires people to perform repetitive finger and foot movements (11). In the later stages of the disease, postural instability is common, resulting in loss of balance and frequent falls (12). Instability is generally absent in the early stages, especially in younger patients, and especially before bilateral symptoms develop (13). Lewy bodies, which are protein inclusions, are another important aspect of PD’s pathology (14). The protein α-synuclein (α-syn) is a major component of Lewy bodies and its mutant forms can cause familial PD (3). The mechanisms that govern α-syn fibrillization and Lewy bodies formation in the brain remain poorly understood.

In contrast to the wealth of information accessible for drug research and development, the use of biomarkers in clinical practice is still underappreciated, and the evidence given in biomarker research for clinical use is still unpersuasive. The same is true of kynurenines (KYNs) and kynurenine pathway (KP) enzymes, which have been linked to a variety of illnesses, such as cancer, autoimmune diseases, inflammatory diseases, neurologic diseases, and psychiatric disorders (15). The tryptophan (TRP)-kynurenine (KYN) metabolic pathway is the main catabolic route of TRP metabolism through which over 95% of TRP degrades into several bioactive metabolites. These metabolites include proinflammatory, anti-inflammatory, oxidative, antioxidative, neurotoxic, neuroprotective, and/or immunologic compounds (16). Alteration in TRP metabolism, glutamate excitotoxicity, and the gut-brain axis have been linked to the etiology of PD (17, 18). While kynurenic acid (KYNA) levels and KYNA/KYN ratios were found significantly lower, the levels of quinolinic acid (QUIN) and ratios of QUIN/KYNA were observed significantly higher in the plasma of PD patients compared to healthy controls in another study (19).

The continual interactions of neurons, glia, and the microenvironment in the central nervous system (CNS) are essential for the preservation of neural homeostasis, and failures in this homeostatic state result in neurodegenerative disorders like PD. The importance of inflammatory processes in the death of dopamine neurons has recently come to light and is now considered as being essential to this process (20–22). The etiology of PD may be significantly influenced by neuroinflammatory processes, according to recent speculation. Numerous research on postmortem, brain imaging, epidemiology, and animal studies have shown that innate and adaptive immunity play a role in neurodegeneration (21). Whether these inflammatory processes are directly responsible for the etiology of PD or are merely subsequent effects of damage to the nigrostriatal pathway is the subject of intensive research. Recently, there has been an increasing emphasis on the identification of mild cognitive impairment (MCI) in PD (PD-MCI) (23), impairments in recognition of emotions (24, 25) deficits in executive functioning, attention, and visuospatial ability, with eventual involvement of memory and other domains (26). It’s interesting to note that the way that PD has been conceptualized over time has evolved from a “motor disease” to a “complex brain disease.” This turnover was supported by the presence of well-documented non-motor disorders, particularly cognitive deficits (23). The pathophysiology of cognitive dysfunction in PD is still up for debate as of this writing. Of late, numerous scholastics believe that cognitive deficits in PD are frequently caused by neuropathological factors such as limbic and cortical Lewy bodies and neurites, amyloid deposition, neurofibrillary tangles, cerebrovascular disease, mitochondrial dysfunction, inflammation, and neurotrophic factors, in addition to neurochemical changes in dopaminergic, cholinergic, and other systems (27).

A number of imaging studies have been conducted in diagnosis of the disease over time such as Computed Tomography (CT) scans (28), voxel-based morphometry (VBM) in group investigations (2, 29–31), manually evaluated predefined region(s)-of-interest (ROI(s) (32) or Computer-aided diagnosis (CAD) approaches (33–35) on an individual basis. Over the last several years, magnetic resonance imaging (MRI) of the brain has been utilized to aid in the diagnosis of PD, with increased detection accuracy (36, 37). We used VBM measurements because of its advanced automation, comprehensiveness, objectivity, and repeatability.

Voxel-based morphometry is a method for calculating differences between two groups of subjects in regional gray matter (GM), white matter (WM), and cerebrospinal fluid (CSF) concentrations by comparing voxel-wise 3-D brain scans (38). To perform VBM, Images from several participants are normalized (contrast stretched) and registered to create a brain template or atlas that reflects a specific set of subjects. The total of 138 PD and 64 age-matched controls MRI images used in this study were obtained from three sites namely Tao Wu (39), Parkinson’s Progression Markers Initiative (PPMI) (40) and NEUROCON (39). The goal of this study was to compare the brains of individuals with PD to controls using the VBM technique.

## Materials and methods

### Datasets

#### Tao Wu

Twenty PD patients (11 males, mean age ± SD 65.2 ± 4.4 years) and 20 age-matched controls (12 males, 64.8 ± 5.6 years) were included in the study. Except for one patient with Hoehn and Yahr stage 3, all patients were in the early to moderate stages of the disease (H&Y stages 1–2.5) (41). The dataset of MRI images are available at the Parkinson’s Disease Datasets^1^ (39).

##### Magnetic resonance imaging acquisition

High-resolution T1-weighted structural images for the 40 individuals were obtained using a Siemens Magnetom Trio 3T scanner (TR = 1100ms, TE = 3.39 ms). MPRAGE images (voxel size 1 × 1 × 1 mm) were also collected for registration to the Montreal Neurological Institute (MNI) template.

#### Parkinson’s Progression Markers Initiative

Data from 91 PD patients (63 males, mean age ± SD 61.3 ± 10.2 years) and 18 age-matched controls (14 males, 64.7 ± 9.7). Patients who have had a diagnosis of PD for 2 years or less and are not on PD medications were included in study. Except for two patients, who were classified as H&Y stage 3, all patients had H&Y scores of 1 to 2. Dataset available at http://www.ppmi-info.org/access-data-specimens/download-data/ (40).

##### Magnetic resonance imaging acquisition

The individuals were scanned in eight separate locations using Siemens Tim Trio 3Tesla scanners with the same protocol (TR = 2.3s, TE = 2.98ms). MPRAGE images (voxel size 1 mm × 1 mm × 1 mm, flip angle = 9°) were also collected for registration to the MNI template.

#### NEUROCON

The NEUROCON study included 27 patients with PD (16 males, mean age ± SD 68.7 ± 10.6) and 16 age-matched normal controls (five males, 67.6 ± 11.9) who had no history of neurological or psychiatric disease. All of the patients were in the early to mid-stages of the disease (H&Y stages 1–2.5).

##### Magnetic resonance imaging acquisition

An MPRAGE sequence was used to acquire high-resolution T1-weighted images for all individuals (IR technique, TR = 1,940 ms, TE = 3.08ms, inversion time (IT) = 1,100 ms, voxel size 0.97 mm × 0.97 mm × 1 mm, number of averages = 1).

In all three sites, 134 out of 138 (97%) patients were in early to mid-stages of the disease (H&Y stages 1–2.5) and only 4 (2.8%) were of stage 3. We decided to use all of the available data for further analysis by combining all patients in one group (90 males, mean age ± SD 63.31 ± 10.06) and controls in another group (31 males, mean age ± SD 65.91 ± 9.65). We used the following formulas to combine mean from three groups.


(1) $${\bar{x}}_{12}=\frac{N_{1}.{\bar{x}}_{1}+N_{2}.{\bar{x}}_{2}}{N_{1}+N_{2}}$$

Where *N_1_* as number of patients in group 1, *N*_2_number of patients in group 2, ${\bar{x}}_{1}$ mean of group 1, ${\bar{x}}_{2}$ mean of group 2 and ${\bar{x}}_{12}$ as combined mean. After getting resultant mean for the two groups. We used the same formula to get combined mean of three groups. See Table 1.

**TABLE 1:** Participant demographics.

Dataset	PD	Controls	PD age	Controls age	Disease duration	H&Y	*P*-value
Tao Wu	20 (11 Males)	20 (12 Males)	65.2 ± 4.4	64.8 ± 5.6	5.4 ± 3.9	1.88 ± 0.63	0.803^a^
PPMI	91 (63 Males)	18 (14 Males)	61.3 ± 10.2	64.7 ± 9.7	1.9 ± 1.0	1.72 ± 0.48	0.196^a^
NEUROCON	27 (16 Males)	26 (5 Males)	68.7 ± 10.6	67.6 ± 11.9	4.6 ± 6.5	1.92 ± 0.33	0.755^a^
Combined	138 (90 Males)	64 (31 Males)	63.31 ± 10.06	65.91 ± 9.65	2.94 ± 3.6	1.78 ± 0.48	0.085^a^

^a^Two-sample *t*-test.

Data are shown in mean ± SD.

We also used the following formula to calculate combined standard deviation (SD) of three groups by first combining two groups and later combining the resultant with the third group.


(2) $$\sigma_{12}=\sqrt{\frac{\left(N_{1}-1\right).\sigma_{1}^{2}+\left(N_{2}-1\right).\sigma_{2}^{2}+\frac{N_{1}.N_{2}}{N_{1}+N_{2}}.\left({\bar{x}}_{1}^{2}+{\bar{x}}_{2}^{2}-2\,{\bar{x}}_{1}\,{\bar{x}}_{2}\right)}{N_{1}+N_{2}-1}}$$

Where *N_1_* as number of patients in group 1, *N*_2_number of patients in group 2, ${\bar{x}}_{1}$ mean of group 1, ${\bar{x}}_{2}$ mean of group 2, σ_1_ SD of group 1, σ_2_ SD of group 2, and σ_12_ as combined SD. We used the same formula to get the combined SD of three groups. See Table 1.

In view of the fact that we combined multicenter data for case-control studies, and this might possibly result in a large variation, we set up the following exclusion criteria to help ensure data quality: (i) subjects with poor structural scans, making successful segmentation unlikely, or without demographic information and (ii) head movement, subjects were excluded if they exceeded the head transition <3 mm, rotation <3°(42). No subject was excluded, whether they were patients or controls, as they all fulfilled the conditions for further analysis. Furthermore, patients from three sites did not differ with respect to sex and age nor regarding all collected clinical variables including age at disease manifestation, disease duration, times of being inpatient and concomitant antipsychotic (all *p*-values ≥ 0.096).

### Voxel-based morphometry

**FIGURE 1:**
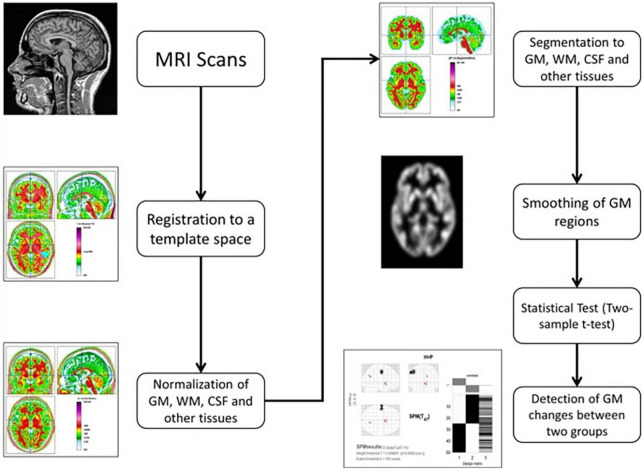
The processing framework of VBM analysis using the CAT12 toolbox of SPM12 software (100).

The CAT12 toolbox, which is included in the SPM12 (43) package, was used for VBM analysis and was run in MATLAB (44). The DARTEL technique was used to spatially normalize and segment all 3D T1-weighted Neuroimaging Informatics Technology Initiative (NIFTI) MR images into GM, WM, and CSF tissue classes using default settings of 1.5 mm cubic resolution in MNI space. The normalized maps were modulated with the resulting Jacobian determinant maps and smoothed with an 8-mm FWHM Gaussian kernel to maintain GM volumes of native space. The operations of segmentation, normalization, and modulation were all performed automatically in the CAT12 toolbox. The total intracranial volume (TIV) was used as a covariate of no interest for estimating the native space volumes of the GM, WM, and CSF maps. The two-tailed *t* test was then produced using family-wise error (FWE) correction and a *p* < 0.05 threshold. The 100 voxel extent threshold was chosen and finally we used *xjview* (45) toolbox for MATLAB to record voxel brain area (represented with pseudo color),with significant differences, activation volume (cluster), activation intensity (statistically analyzed with t-test and expressed as T value; T value is proportional to the intensity). Figure 1 depicts the VBM analysis processing framework.

## Results

### Participants’ demographic data

Table 1 summarizes participants’ demographic information. In which there was no statistically significant difference between PD patients and controls with respect to sex and age nor regarding all collected clinical variables including age at disease manifestation, disease duration, times of being inpatient and concomitant antipsychotic (*p* > 0.05) using two-sample *t*-test. The combined data were of 138 PD patients (90 males, mean age ± SD 63.31 ± 10.06 and 64 age-matched controls (31 males, 65.91 ± 9.65). Combined disease duration 2.94 ± 3.6 years and H&Y scores 1.78 ± 0.48. Gender was analyzed by chi-square test; other variables were analyzed by independent samples *t*-test. The three groups of patients were well matched in age, gender and disease duration.

### The voxel-based morphometry analysis

**FIGURE 2:**
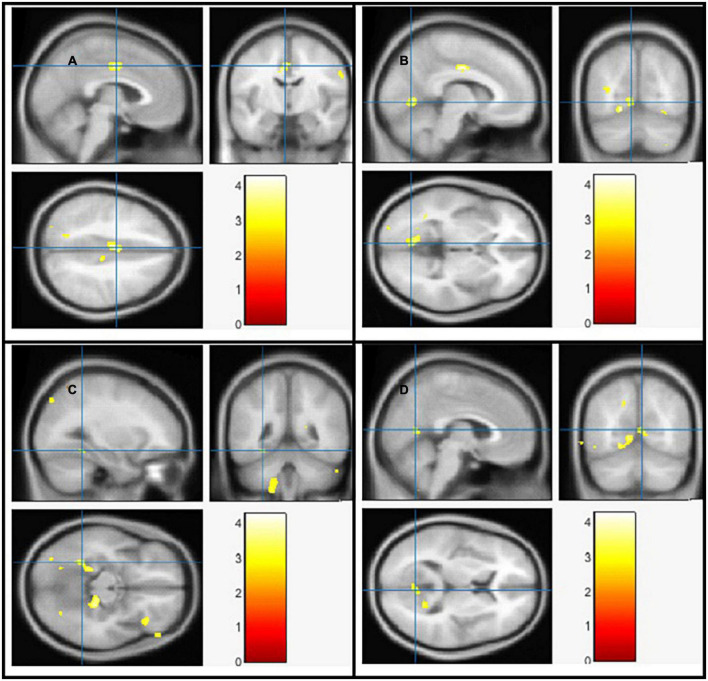
The significant gray matter (GM) alterations by VBM analyses with the covariate of no interest (TIV) in the left middle cingulate **(A)**, left lingual gyrus **(B)**, left fusiform gyrus **(C)**, and right calcarine **(D)**, respectively when PD < controls with *p* < 0.05 and extent threshold *K* = 100.

**FIGURE 3:**
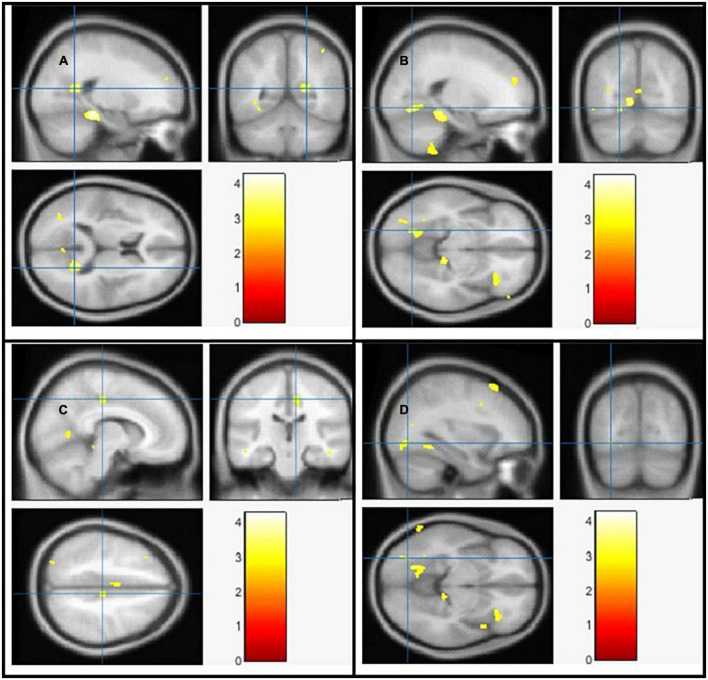
The significant white matter (WM) alterations by VBM analyses with the covariate of no interest (TIV) in the right calcarine **(A)**, left lingual gyrus **(B)**, right middle cingulate **(C)**, and left inferior occipital gyrus **(D)**, respectively when PD < controls with *p* < 0.05 and extent threshold *K* = 100.

**FIGURE 4:**
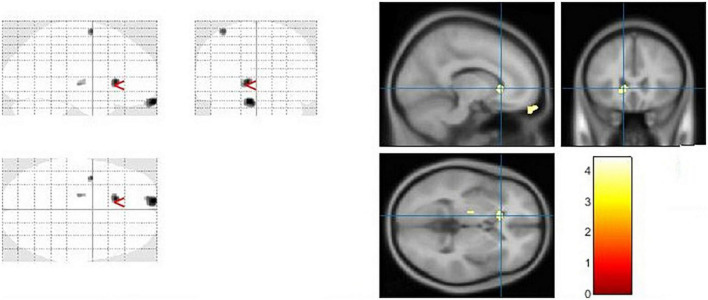
The significant cerebrospinal fluid (CSF) alterations by VBM analyses with the covariate of no interest (TIV) in the left caudate when PD > controls with *p* < 0.05 and extent threshold *K* = 100.

Using Family-Wise Error (FWE) with *p* < 0.05 in the *t* test in voxel by voxel analysis, four locations; left middle cingulate, left lingual gyrus, left fusiform gyrus and right calcarine in the PD participants had lower GM ratios than the HC subjects also WM loss was found extensively in the right calcarine, left lingual gyrus, right middle cingulate, and left Inferior occipital gyrus. Moreover, PD patient group demonstrated higher CSF in the left caudate compared to the controls Figures 2–4 and Tables 2–4 show the relevant regions and MNI coordinates of the peak voxels. It should be indicated that when the contrast, PD > controls subjects was selected, no brain regions exhibited significant GM (Table 2) or WM (Table 3) alterations in the patients over the controls, but exhibited significant CSF (Table 4) alterations in the patients over the controls.

**TABLE 2:** Gray matter alterations detected by VBM.

*P*-value	Contrast	Anatomical region	L/R	Size (Voxels)	No. of clusters	MNI coordinates (mm)			Voxel level	
						*X*	*Y*	*Z*	*T*-value	*Z*-value
*p* < 0.05	PD < C	Middle cingulate	L	136	10	–3	–8	41	4.3	3.85
		Lingual gyrus	L	58	7	–8	–69	–2	3.72	3.41
		Fusiform gyrus	L	15	4	–29	–47	–14	3.63	3.34
		Calcarine fissure	R	5	8	5	–65	9	3.37	3.12
	PD > C	–	–	–	–	−	−	−	–	–

PD, Parkinson’s disease; C, controls; L, left; R, Right; MNI, Montreal Neurological Institute.

**TABLE 3:** White matter alterations detected by VBM.

*P*-value	Contrast	Anatomical region	L/R	Size (Voxels)	No. of clusters	MNI coordinates (mm)			Voxel level	
						*X*	*Y*	*Z*	*T*-value	*Z*-value
*p* < 0.05	PD < C	Calcarine fissure	R	98	9	23	–54	12	4.09	3.69
		Lingual gyrus	L	24	5	–21	–68	–9	3.99	3.61
		Middle cingulate	R	11	11	9	–23	45	3.49	3.22
		Inferior occipital gyrus	L	8	6	–32	–75	–8	3.61	3.32
	PD > C	–	–		–	−	−	−	–	–

PD, Parkinson’s disease; C, controls; L, left; R, right; MNI, Montreal Neurological Institute.

**TABLE 4:** Cerebrospinal fluid alterations detected by VBM.

*P*-value	Contrast	Anatomical region	L/R	Size (Voxels)	No. of clusters	MNI coordinates (mm)			Voxel level	
						*X*	*Y*	*Z*	*T*-value	*Z*-value
*p* < 0.05	PD < C	–	–	–	–	–	–	–	–	–
	PD > C	Caudate	L	134	8	−12	24	0.00	4.23	3.79

PD, Parkinson’s disease; C, controls; L, left; R, right; MNI, Montreal Neurological Institute.

## Discussion

The goal of our research was to see if there were any anatomical differences in the brains of people with PD and controls. We compared the PD patients to the controls in Tables 2, 3 using a two-tailed *t* test with a covariate of no interest (i.e., TIV), *p* < 0.05 and extent threshold *K* = 100. According to our findings, while the PD patients showed significant clusters of reduced GM volumes in the left middle cingulate, left lingual gyrus, left fusiform gyrus, and right calcarine, they also showed reduced WM volumes in right calcarine, left lingual gyrus, right middle cingulate and left inferior occipital gyrus. Moreover, PD patient group demonstrated a higher CSF in the left caudate compared to the controls.

### Gray and white matter atrophy in cingulate gyrus

The Cingulate gyrus is located on the medial aspect of the cerebral hemisphere, a fundamental component of the limbic system, which regulates emotion and behavior (46). There were significant reduction of GM and WM in left middle cingulate and right middle cingulate respectively in brains of PD patients compared to the controls. To compare our findings with other studies, a number of highly expressed genes in the cingulate networks were linked to diminished GM integrity in PD, according to one study (47), another study demonstrated that cognitive impairment and excessive daytime sleepiness were associated with atrophy in cingulate network in PD (48). Goldman and colleagues (49) revealed GM atrophy in cingulate among other brain parts in PD patients with visual hallucinations compared to non-hallucinators. WM reduction was also found in the cingulate, among other locations using Network Based Statistic (NBS) analysis in PD patients compared to young and middle-aged healthy subject groups (50). While Vercruysse and colleagues (51) demonstrated anatomical abnormalities in the cingulate cortex of PD patients utilizing both WM and GM. However, the results are inconsistent among other studies for example, greater GM volume was found in the anterior cingulate cortex in PD patients compared to controls (52), the inconsistencies could be due to research diversity, methodologic differences, and patient sample size differences. Moreover, loss of cingulate GM/WM volume was associated with other illness/disorders i.e., internet gaming disorder (53), bipolar disorder (54–56), and schizophrenia (57).

### Gray and white matter atrophy in occipital lobe (lingual gyrus, calcarine, and fusiform gyrus)

The occipital lobe houses the majority of the visual cortex in the brain, allowing us to view and interpret external inputs as well as assign meaning to and retain visual sensations. Our neuroimaging findings are consistent with earlier VBM investigations in PD patients that have found a link between cognitive impairment and GM loss in the occipital lobes, particularly in PD patients with dementia (PDD) (2, 58) and PD patients with mild cognitive impairment (MCI) (59). In 2009, a VBM investigation by Pereira and colleagues revealed that, patients with PD who did poorly on visuospatial/visuoperceptual tests had GM cortical loss in the parietal and occipital areas (60). More analysis revealed loss of GM in the bilateral orbitofrontal and right temporal areas as well as the limbic system was linked to depression in PD (61). In another interesting study on identifying structural candidates according to cognitive status in PD, GM density was considerably lower in the left occipital area in PD-intact cognition (PD-IC) and the right occipital area in mild cognitive impairment in PD (PD-MCI) compared to controls (62) also reduced GM volume in the lateral occipital cortex was linked to cognitive impairment and physical frailty in another study (63).

The primary visual cortex is situated in the calcarine region of the occipital lobe in which previous research has proven the link between visuospatial skills and motor function (64). In our work we found reduction of both GM and WM in the right calcarine. In line with other studies, lower GM volume in the left calcarine and right inferior frontal gyrus was linked to the higher risk of falling (65) especially in patients with PDD. We also found reduction of both GM and WM in the left lingual gyrus, as a brain structure involved in visual processing, particularly in relation to letters and logical condition analysis, other study also reported the association of Freezing of Gait (FOG+) with the reduction of GM volume in lingual gyrus compared with both patients with FOG- and HCs (66) based on their responses to a validated FOG questionnaire and clinical observation.

The fusiform gyrus has been linked to high-level visual processing activities, including the processing of information about faces, bodies, and stimuli with high spatial frequency (67). Consistent with our work were we found the reduction of GM in the left fusiform of the PD group, other VBM analysis found GM reductions in the PD-MCI group, especially in fusiform gyrus among other areas compared to PD without MCI and controls (59), another study linked GM reduction in fusiform gyrus to poor visuoperceptual performance (60). Patients with PD had lower baroreflex sensitivity (BRS) versus controls, indicating poor cardiovascular autonomic function, and reduced GM volume in multiple brain areas, including the right fusiform, which was linked to an increased presence of epithelial progenitor cells (EPCs) in the circulation (68). Reduced effective connections in the fusiform gyrus have been associated with body size misjudgment score in studies, suggesting that these areas may play a role in the development of anorexia nervosa (69).

However, one study reported increased GM volume in occipital areas in PD patients compared to the controls (52). Although, to the best of our knowledge, there is no evidence of the mechanisms causing increased GM volume in PD, which is a continuously progressing neurodegenerative condition, the impact could represent a compensatory mechanism to impaired brain function in early PD (52, 70). WM atrophy using VBM analysis was also observed in occipital area according to the previous reports, such as visual hallucinations both in PD (71), schizophrenia (72–74), mood and anxiety disorders (75–77) and association of imbalance in the ratio of WM fibers in occipital lobe with psychosis in PD (78). Auning et al. using diffusion tensor imaging (DTI) when compared to controls, patients with PD demonstrated substantial changes in WM underlying the temporal, parietal, and occipital area (79). These study demonstrate that WM affection is linked to the cognitive impairment in PD, and that brain changes occur in a sequential pattern, with hypoperfusion coming first, followed by WM damage and GM atrophy (80). Although there is some discrepancy across these studies, several contributing factors, such as number of cases, clinical features, and disease severity may contribute to the differences. Our findings reveal that PD patients have more GM and WM loss than controls, particularly in the cingulate gyrus and occipital lobe. Detecting these specific anomalies can help with PD diagnosis. It have been reported that mindfulness-based intervention (81, 82), stress management training (83) and Mind-Body technique (relaxation guided imagery) (84, 85) may help in the alleviation of both motor and non-motor symptoms as well as the slowing of disease progression.

### Higher cerebrospinal fluid in the caudate nucleus

The caudate nucleus is a component of the basal ganglia (nervous system). The basal ganglia are a collection of subcortical nuclei that play a variety of cognitive and affective roles, but are best known for their role in movement. However, the caudate is assumed to be involved in more than only motor function. A neuroimaging study (86), for example, have suggested that the caudate has a role in goal-directed behavior in general. Our current study, reveals some intriguing CSF findings in which PD patients exhibited higher CSF in the left caudate compared to the controls, to the best of our knowledge, this is the first demonstration to link PD with increased CSF in the caudate. CSF is a transparent fluid that circulates across the intracranial and spinal compartments. The composition of CSF remains consistent under normal circumstances. However, the quantity, content, and pressure of the fluid can be altered in numerous neurological diseases, particularly in PD and other conditions. For example unilateral and bilateral choroid plexus papilloma’s have been linked to increased CSF production (87). The lack of underlying conditions that caused the patient’s CSF overproduction, as well as comorbidities, posed unique challenges in this study.

Understanding the structural alterations causing cognitive deterioration in PD is therefore crucial for early diagnosis and providing effective treatment. Here is the summary of the neuroanatomical changes in major brain structures responsible for cognition in PD. (i) Changes in brain volume in PD; cognitive abilities and brain size are strongly correlated (88). Many cortical and subcortical parts of the brain in PD patients showed signs of shrinkage, which contributed to a reduction in the brain’s volume (89). It’s interesting to note that PD patients were found to have increased volume in their frontal lobe, temporoparietal junction, parietal lobe, insula, anterior cingulate cortex, basal ganglia, and thalamus (90). Biundo et al. reported that prefrontal lobe is essential for cognitive processes and is affected by GM loss in PD patients (91). (ii) Changes in basal ganglia in PD; the basal ganglia are important for cognitive processes, and their damage impairs cognitive processes. In reality, the most damaged part of the brain in PD is the basal ganglia (89). According to the previous literature, there are two subtypes of PD, and each type has a unique impact on the basal ganglia. The subtypes include several clinical manifestations, such as postural instability/gait difficulties (PIGD) and tremor dominant (TD) (92). It has also been reported that reduction in the volumes of the caudate nucleus and thalamus and WM is observed in PD, which may be an early sign of disease progression (93). (iii) Changes in cerebellum in PD; basal ganglia and the cerebellum are connected reciprocally, and PD-related morphological abnormalities have been observed in both animal models and humans (94). The left cerebellum has seen a substantial contraction, while the right quadrangular lobe’s GM volume has decreased. These alterations might be brought on by degeneration of dopaminergic neurons (95). (iv) Changes in thalamus in PD; language, memory, and attention have all been reported to suffer from thalamic lesions. Thalamic stimulation was effective in enhancement of cognition through activation of neocortex and hippocampus and modulating gene expression (96). In addition, depression affects the majority of PD patients due to abnormalities in the WM of the mediodorsal thalamus (97). (v) Changes in hypothalamus in PD; dopamine dysfunction in the hypothalamus may contribute to the emergence of sleep, endocrine, and autonomic abnormalities in PD. Given that melatonin levels are linked to the volume of GM, it has been reported that sleep disorders may be associated with hypothalamic GM loss (98) and (vi) Changes in limbic system in PD; changes in creativity and emotional dysfunction in PD patients have been linked with dopamine dysfunction in the limbic system (99). Apart from the said neuroanatomical changes in major brain structures responsible for cognition in PD, in this paper we believe that physical fragility and cognitive impairments in PD may be detected more easily if anatomical abnormalities to the cingulate gyrus, occipital lobe and the level of CSF in the caudate are identified.

## Limitations and future directions

However, there are some drawbacks to this study. The lack of clinical assessments associated to PD prevented us from assessing potential links between these patterns of abnormalities identified in PD and clinical evaluations. Moreover, because the patients were selected from three centers (Tao Wu, PPMI, and NEUROCON), they may not be representative to all PD population. Future research may require to clarify the causal links between GM and WM volumes, cognitive decline, and physical frailty. To validate our findings, more research combining neuroimaging, biochemistry, and clinical assessments are needed.

## Conclusion

Using VBM, we explored structural brain differences between group of 138 PD patients and 64 healthy people. PD patients exhibited reduced GM and WM volumes versus controls especially in the cingulate gyrus and occipital lobe and increased CSF in the left caudate. Thus, physical fragility and cognitive impairments in PD may be detected more easily if anatomical abnormalities to the cingulate gyrus, occipital lobe and the level of CSF in the caudate are identified. Previous literatures suggest that mindfulness-based intervention, stress management training and relaxation guided imagery may help in the alleviation of both motor and non-motor symptoms and slow the disease progression. In this study, we have shown how important cognition-related brain areas changed neuroanatomically in PD. We have also incorporated the major findings of numerous studies in order to provide current information for a better understanding of the pathophysiology of PD, which aids researchers and clinicians in planning and developing new treatment approaches for the benefit of PD patients. Although we cannot fully attribute any possible differences to the effects of GM, WM, and CSF between the groups due to the lack of clinical data, we believe that neuroimaging results in this study correspond with the clinical presentation as well as the cognitive changes. This hypothesis will need to be investigated in the future as well as integrating VBM with functional techniques such as functional MRI and EEG/MEG to better define the links between brain function and structure in health and disease.

## Data availability statement

Publicly available datasets were analyzed in this study. This data can be found here: http://fcon_1000.projects.nitrc.org/indi/retro/parkinsons.html and http://www.ppmi-info.org/access-data-specimens/download-data.

## Author contributions

CN, LQ, MA, and HK: substantial contributions to the conception or design of the work and contributions to the acquisition, analysis, or interpretation of data, and drafting the work or revising it critically for important intellectual content. CN and LQ: final approval of the version submitted. All authors contributed to the article and approved the submitted version.

## Footnotes

1
http://fcon_1000.projects.nitrc.org/indi/retro/parkinsons.html
